# Addressing Information Gaps and Revising Coverage Terms and Conditions for Cancer Panel Testing

**DOI:** 10.31662/jmaj.2024-0014

**Published:** 2024-04-01

**Authors:** Yasuhito Nannya

**Affiliations:** 1 Division of Hematopoietic Disease Control, Institute of Medical Science, The University of Tokyo, Tokyo, Japan

**Keywords:** Clinical genomics, cancer genome panel, information gap

Efforts in genomic medicine in Japan have historically lagged behind those in other countries. However, through the Council for the Promotion of Genomic Medicine, established under the Health and Medical Care Strategy Promotion Council of the Cabinet Secretariat, ministries and agencies have been collaborating to make genomic medicine a practical reality. As a result, two “cancer gene panel tests” were approved for insurance coverage starting in June 2019, marking the beginning of genomic medicine in Japan. In the 4 years since then, approximately 20,000 patients have undergone cancer gene panel testing annually (Center for Cancer Genomics and Advanced Therapeutics (C-CAT) registration number from January to December 2022: 1,400-1,900 patients/month). However, compared with the annual number of cancer deaths (approximately 380,000), the percentage of patients who underwent genome tests is low. The primary reason for this may lie in the purpose and implementation of the cancer gene panel tests. Currently, cancer gene panel tests that are covered by insurance are intended for patients with solid tumors lacking standard treatment options or those with locally advanced or metastatic disease for which standard treatment has been completed (including expected completion). In addition, cancer gene panel testing is limited to 13 cancer genomic medicine core base hospitals, 32 cancer genomic medicine core hospitals, and 215 cancer genomic medicine cooperative hospitals nationwide. In these hospitals, the genomic testing results of each case are reviewed by an expert panel to determine the presence or absence of pathogenicity, secondary findings, or other results that require special attention. However, the considerable preparation time and human costs associated with expert panels have prevented an increase in the number of tests. While these limitations may be necessary to appropriately allocate finite medical resources, any information gaps among healthcare providers preventing the implementation of cancer panel tests for patients who need them must be promptly rectified. A survey conducted by Uezaki et al. among 14,579 physicians at Japanese Board of Cancer Therapy-accredited facilities revealed institutional differences in knowledge, experience, and the actual implementation of cancer gene testing ^(1)^. Although differences in genomic medicine experience between institutions are expected, the survey highlighted significant information gaps, suggesting that physicians may struggle to refer patients who could benefit from cancer panel testing to suitable facilities that can perform the test due to lack of knowledge. This is extremely important in Japan’s insurance system, which provides equal healthcare nationwide. Conversely, the survey showed few regional differences in the experience and knowledge of genomic medicine. Although it is necessary to travel to the core cities of the region, genomic medicine can be provided in any rural area. While various educational programs exist to address information gaps in genomic medicine, there is a need to add a section promoting medical collaboration to these programs. It would be desirable not only to provide an understanding of the simple purpose and methods of cancer gene panel testing but also to develop and advertise the existence of an information portal where people can quickly search for a list of facilities to which they can be referred and how to make referrals.

The largest barrier to widespread use of genomic tests is the fact that their purpose is extremely limited. The purpose of the current genome test is to identify gene mutations in patients who have completed standard treatment and are either not receiving standard treatment or have locally advanced or metastatic disease, with the aim of offering treatment, including molecularly targeted drugs. However, the proportion of patients who receive treatment as a result of testing is currently low. The Cancer Genome Information Center (C-CAT) reported that 8.1% of patients received new treatment as a result of undergoing cancer gene panel testing, and the most recent report in February 2023 indicated that 9.4% of patients received new treatment ^(2)^. These numbers do not make this test attractive. These comments have also impacted patient satisfaction, with approximately 80% of all groups―patients/family members, the general public, and healthcare professionals―stating in the public seminar questionnaire that they would like the test to be available at the stage of cancer diagnosis. It is reasonable to limit the number of eligible patients and carefully manage the test at the beginning. However, >4 years have passed since the start of the program, which warrants a review of the system. Particularly, coverage should be expanded to include cancer gene panel testing at the pretreatment stage for advanced or recurrent solid tumors that are expected to be difficult to cure. A study by Kyoto University found that the percentage of patients who received recommended treatment was three times higher when CGP was performed before standard treatment (approximately 19.8%) than after its completion ^(3)^. Furthermore, early implementation of CGP in solid tumors in general requires verification of its usefulness from multiple perspectives, diagnosis, prognostic including prediction, drug sensitivity, response to secondary findings, and patient satisfaction, in addition to improving drug efficacy.

## Article Information

### Conflicts of Interest

None
